# Mass spectroscopy reveals compositional differences in copepodamides from limnic and marine copepods

**DOI:** 10.1038/s41598-024-53247-1

**Published:** 2024-02-07

**Authors:** Sina Arnoldt, Milad Pourdanandeh, Ingvar Spikkeland, Mats X. Andersson, Erik Selander

**Affiliations:** 1 Department of Marine Sciences, University of Gothenburg, Medicinaregatan 7B, 41390 Gothenburg, Sweden; 2 Department of Haldenvassdragets Kanalmuseum, Østfold Museum Foundation, Gamlebygata 8, 1721 Sarpsborg, Østfold Norway; 3 Department of Biological and Environmental Sciences, University of Gothenburg, Medicinaregatan 7B, 41390 Gothenburg, Sweden; 4 Department of Biology-Aquatic Ecology, Lund University, Sölvegatan 37, 22362 Lund, Sweden

**Keywords:** Ecology, Limnology

## Abstract

Marine copepods, the most abundant animals in the global ocean, imprint their surrounding waters with chemical cues, called copepodamides. Copepodamides induce defensive traits such as toxin production, bioluminescence, and colony size plasticity in a variety of marine phytoplankton. The role of copepodamides in freshwater ecosystems is, however, unknown. Here we report the consistent presence of copepodamides in copepods from six Swedish freshwater lakes. Copepodamide concentrations in freshwater copepods are similar to those of marine copepods, around 0.1 ppt of dry mass in millimetre sized individuals. The composition substantially overlaps with marine copepodamides but is also distinctly different. Marine copepods commonly contain both subgroups of copepodamides, the copepodamides (CA) and the dihydro-copepodamides (dhCA), whereas freshwater copepods are dominated by the dhCAs. Taxonomic groups had consistent copepodamide profiles across sampling sites and timepoints, supporting the presence of species-specific copepodamide signatures. We describe 10 new copepodamide structures, four of which were found exclusively in freshwater copepods. The presence of copepodamides in limnic copepods also warrants studies into their potential function as predator alarm cues in freshwater systems.

## Introduction

**Figure 1:**
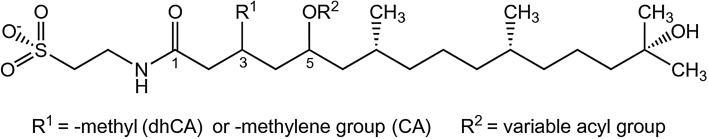
General structure of copepodamides. Two main subgroups exist, determined by the presence of methylene (copepodamide/CA) or methyl (dihydro-copepodamide/dhCA) at R^1^. The blend is species specific, but the fatty acid side chain (at position R^2^) changes with diet. Copepodamides are named by the acyl group^8^ followed by the scaffold name e.g. 22:6 dihydro-copepodamide for a dhCA scaffold with a docosahexaenoic acyl group in position R^2^.

Phytoplankton sense chemical alarm cues from crustacean zooplankton predators and respond by inducing a range of defensive traits^1,2^. These include morphological, behavioural, chemical, and life history responses^3–6^. Two groups of cueing compounds have been identified so far, aliphatic sulphates and copepodamides. Aliphatic sulphates (or sulfamates) are a group of short chain (9–11 carbon) aliphatic compounds in freshwater cladocerans, sometimes branched and with different levels of saturation, that induce colony formation in the freshwater green algae *Desmodesmus subspicatus* (previously *Scenedesmus subspicatus*)^7^. Marine copepods produce the other known class of defence inducing compounds, copepodamides, a group of structurally closely related taurine conjugated lipids (Fig. 1). Copepodamides have been found in all sampled marine cyclopoid and calanoid copepods^8,9^, with the possible exception of carnivorous species^10^. Given the dominance of copepods in all oceans, copepodamides are likely among the most widespread chemical alarm cues known. Copepodamides, hereafter denoting the general group of compounds, are further divided into two subgroups based on the presence of a methyl (dihydro-copepodamides, hereafter “dhCA”) or methylene group (copepodamides, hereafter “CA”) in position C3 (Fig. 1). The fatty acid attached to C5 is variable and changes with diet^8^. Long chain polyunsaturated ω-3 fatty acids such as docosahexaenoic (22:6), eicosapentaenoic (20:5), or stearidonic (18:4) are common in marine copepodamides^9^. Other fatty acids, however, including even-numbered saturated fatty acids such as myristic (14:0) or palmitic (16:0) acid also occur. Thirty-one copepodamide derivatives have been described to date^8^. Copepodamide concentrations in the ocean are correlated to copepod densities in the local environment and reach bioactive levels when copepods are abundant^11^. Harmful algal bloom (HAB) forming phycotoxin producers such as *Pseudo-nitzschia* spp. and *Alexandrium* spp. respond by producing more amnesic^10^ and paralytic shellfish toxins^9^. Bioluminescent taxa such as *Lingulodinium polyedrum* and *Alexandrium tamarense* increase light production^12^. Chain forming diatoms split up colonies into smaller units^13,14^. Other diatoms have been shown to increase silica content and stickiness, which results in cell aggregation^15^. Induced toxin production and morphological changes are accompanied by increased resistance to grazers^14,16,17^. Well defended taxa may subsequently benefit from a competitive edge which can contribute to the formation of HABs. Incorporating copepod densities, or direct measurements of copepodamides in mussels, have been suggested to improve the lead time and precision in HAB forecasting models^18^.

Aliphatic sulphates have only been reported from limnic environments, whereas copepodamides have, except for a single measurement in a Swedish pond^8^, only been found in marine copepods. There are, however, empirical evidence that limnic dinoflagellates also respond to chemical cues from copepod grazers. Resting stages of freshwater dinoflagellates of the genera *Ceratium* and *Peridinium* delay excystment when copepods are abundant in the water column above^19^. The identity of the cueing compound(s) is not known, and the role of copepodamides in limnic ecosystems remains to be explored.

Here we prove the presence of copepodamides in freshwater copepods from five lakes located on the Swedish west coast and one northern latitude ice-covered lake. We analyse bulk zooplankton samples and extracts from individual copepods to compare the composition and amounts of copepodamides between freshwater and marine copepods. We find that freshwater copepods also contain copepodamides, and that similarly sized copepods contain comparable amounts of these regardless of habitat. We also prove that the composition of copepodamides in freshwater copepods is distinctly different from marine ones.

## Materials and methods

### Collection of copepods

**Figure 2:**
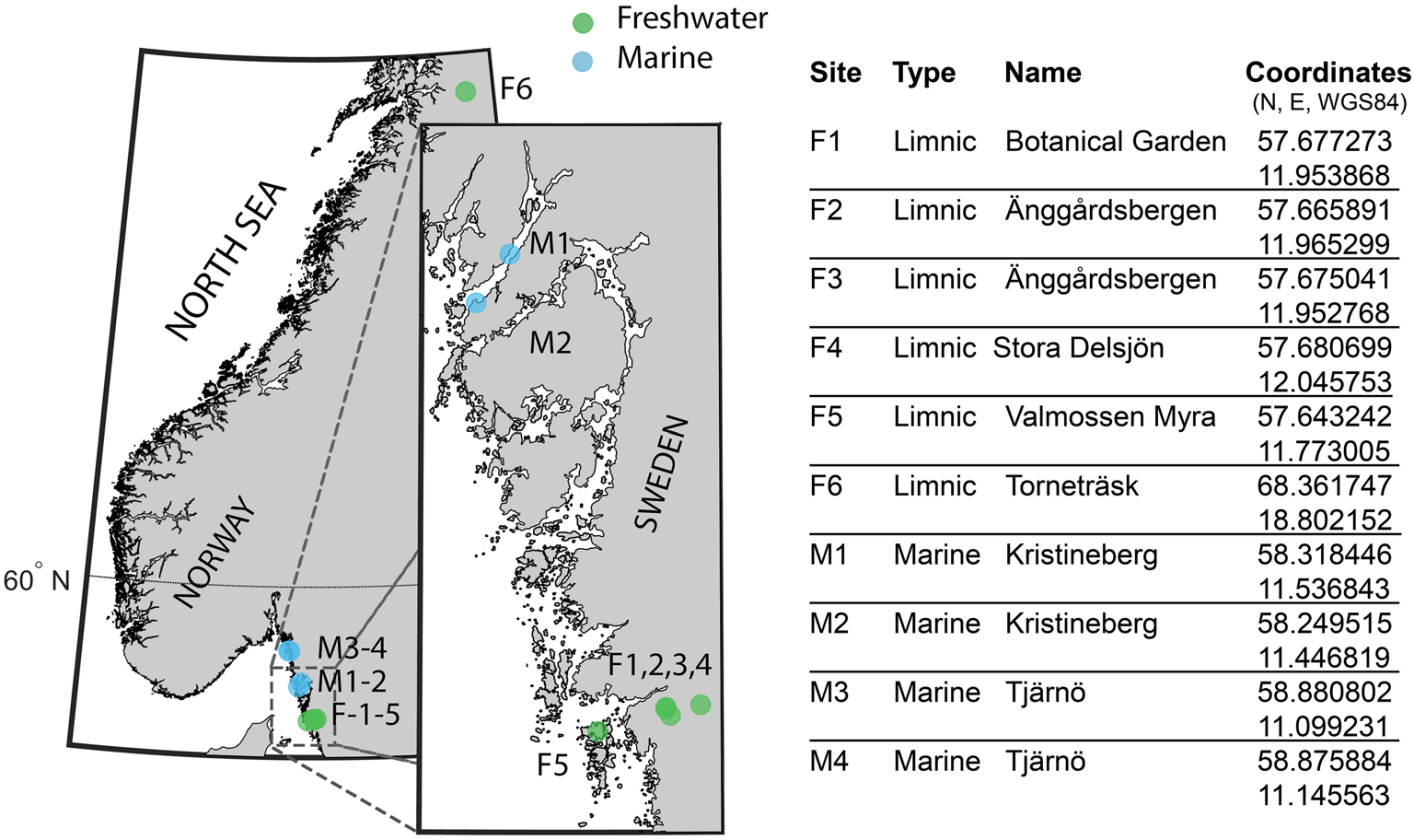
Map of sampling stations (left) with names and positions (right table). Freshwater sites are shown in green and marine in blue. Stations F1–F3 markers overlap, F5 is located on the island Brännö. M3 and M4 markers overlap and are in the Koster Fjord. Maps produced in MATLAB 2023a.

We sampled six lakes/ponds and four marine sites (Fig. 2, see Supplementary Table S1 for detailed information about the sampling sites). Freshwater zooplankton were collected with a handheld plankton net (mesh size: 65 μm, diameter: 15 cm), except for the ice-covered lake (F6) which was sampled through the ice with a smaller net (mesh size: 25 µm, diameter: 10 cm). Marine zooplankton were sampled with the same handheld plankton net at sampling sites M2 and M4, with a WP2 net (mesh size: 90 µm, diameter 57 cm diameter) onboard R/V Oscar von Sydow at M1, and with a handheld net (mesh size 200 µm, diameter: 57 cm) from a small outboard powered boat at M3. Horizontal/oblique net tows at 1–2 m depth were carried out at all sampling stations, except for M1, M3, and F6. We used vertical tows at M1 and M3, from 25 and 50 m depth to the surface, respectively. Vertical tows were also used to sample through the ~ 60 cm diameter ice-hole at F6. The zooplankton samples were brought back to the lab alive, in water from the sampling site, and extracted as quickly as possible (0.5–7 h, median = 2.5 h).

From each of the 10 bulk zooplankton samples, we transferred 9–23 adult copepods or late stage copepodites (116 individuals in total, Supplementary Table S2) into individual 1.5 mL HPLC glass vials. We immediately removed the water from these with a Pasteur pipette, added 1 mL methanol and left the copepods to extract overnight at − 20 °C. The extracts were then transferred to glass tubes and concentrated by evaporation until dry under a stream of nitrogen (40 °C) and re-dissolved in 50 μL methanol before storage at − 20 °C until analysis. We identified the copepods to their lowest possible taxonomic level and measured their prosome length under an inverted microscope (Axiovert A1, Zeiss). Biomass (dry weight) was then estimated from collated prosome length–weight regressions (Supplementary Table S3). The remaining bulk zooplankton samples were extracted as described for individual copepods above to generate more concentrated samples.

### Copepodamide analysis

We analysed the copepodamides on an Agilent 1260 HPLC system coupled to an Agilent 6475 triple quadrupole MS detector. The copepodamides were separated on a Prevail C18, 3 μm, 2.1*150 mm column thermostated to 50 °C using a gradient elution from 95% eluent A (methanol: acetonitrile: water; 35:35:30) and 5% eluent B (isopropanol) to 83% B over 18 min followed by a 7 min re-equilibration. 0.2% formic acid and 0.1% ammonia (v/v) was added to both eluents before chromatography. Bulk zooplankton samples were scanned for compounds of 600–1000 m*/z* producing the product ions characteristic of CAs (430.3 m*/z*) or dhCAs (432.3 m*/z*, Supplementary Data 1). The most abundant of these transitions, making up ≥ 80% of the total ion counts, were combined with the previously known copepodamides^8^ for multiple reaction monitoring (MRM) experiments on individually extracted copepod samples (Supplementary Data 2). We calibrated copepodamide measurements against authentic copepodamide standards isolated from marine copepods (*Calanus finmarchicus*), assuming the same ionisation efficiency for all copepodamides. For detailed information on MS settings and preparation of the analytical standard, see Selander et al.^9^.

### Multivariate ordinations and statistical analyses

We compared copepodamide composition of marine and freshwater samples with permutational multivariate analysis of variance (PERMANOVA)^20^ using the R package *vegan*^21^. Homogeneity of variance was tested with a permutation test of multivariate dispersion (PERMDISP)^22^. Non-metric multidimensional scaling (nMDS) was used to visualise the multivariate data in two-dimensional space. We tested for differences in copepodamide content between the individually sorted freshwater and marine copepods with analysis of covariance (ANCOVA), controlling for their body size (estimated dry mass). Assumptions of (1) linearity were assessed both visually and with linear regression, (2) homogeneity of regression slopes by evaluating the covariate-predictor interaction, and (3) conditional normality was both assessed visually and formally tested with Shapiro-Wilks’ test. The data was non-normally distributed, but ANCOVA (and ANOVAs in general) procedures are robust against violations of normality when sample sizes are large and similar among groups^23^. We used Bray–Curtis distance^24^ as the dissimilarity metric for all nMDS, PERMANOVA and PERMDISP procedures, and set permutations to n = 9999 and the significance level (ɑ) to 0.05 for all tests. All statistical analyses were performed in R version 4.2.3^25^ via the RStudio IDE^26^.

## Results

**Figure 3:**
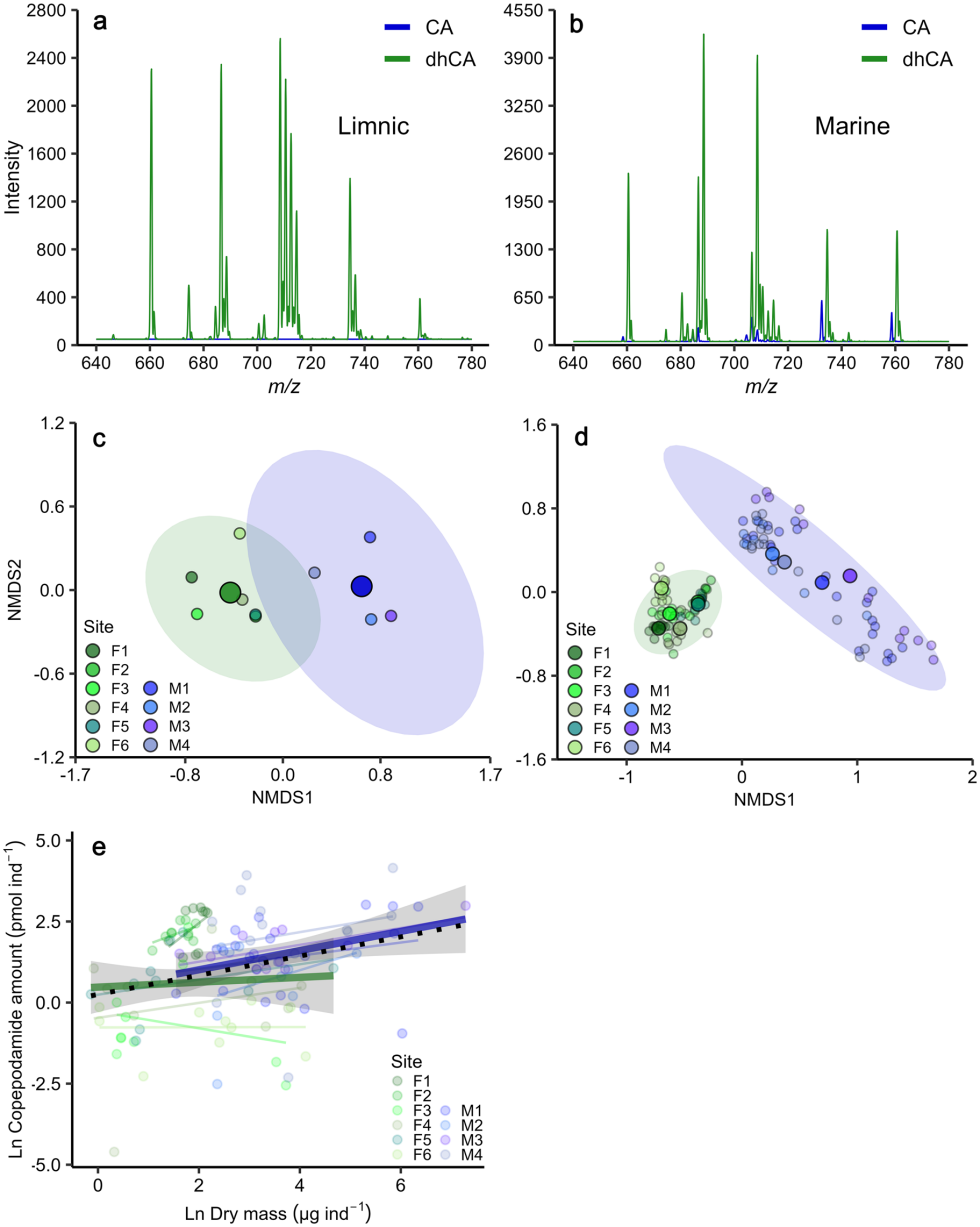
Copepodamide composition differs between marine and limnic copepods, both in bulk samples and in individually analysed copepods, but the size normalised content is similar. (**a**) Representative mass spectra from precursor ion scans of copepodamides in bulk zooplankton samples from limnic (F5) and (**b**) marine (M4). Note the absence of CA (blue) in the limnic samples. **c**: Non-metric dimensional scaling (nMDS) ordination of copepodamide composition in bulk zooplankton samples for each site (smaller points) and (**d**) in individual copepods (smaller points). Larger points denote the centroid for each group (i.e. habitat type average in **c** and sampling site average in **d**), coloured ellipses are 95% confidence intervals of centroids. Stress values for the nMDS models were 0.04 and 0.11 respectively. (**e**) Total copepodamide content in individual copepods (pmol ind^−1^, filled circles) plotted against their estimated dry mass (µg). Thin lines denote regression lines for the individual sampling sites, thicker coloured lines denote regression lines for the two habitat types and the dotted black line denotes the global regression (Ln(CA pmol) = 0.296 * Ln(dry mass µg) + 0.25, R^2^ = 0.087). Shaded error bands denote 95% confidence intervals for the habitat regressions.

The screening of bulk zooplankton samples revealed the presence of diverse copepodamides in both freshwater and marine samples. In total we detected 35 putative copepodamide structures, six of which were exclusively present in freshwater copepods, 18 only in marine, and 11 in both marine and freshwater copepods (Fig. 3a, b, Table 1). Fragmentation experiments of abundant compounds confirmed the presence of fragments associated with dhCA (C_22_H_42_NO_5_S; *m/z* 432) and CA scaffolds (C_22_H_40_NO_5_S; *m/z* 430), as well as fragments matching taurine (C_2_H_6_NO_3_S; *m/z* 124.0) and sulfonate (SO_3;_ *m/z* 80). The composition of copepodamides differed significantly between freshwater and marine bulk zooplankton samples (PERMANOVA: df = 1, 8, p = 0.004, Fig. 3c). The targeted analysis (MRM) of individually extracted copepods confirmed this difference (PERMANOVA: df = 1, 102, p < 0.001, Fig. 3d) and unequivocally shows the presence of copepodamides in freshwater copepods.

**Table 1:** List of putative copepodamides in freshwater and marine bulk zooplankton samples.

Scaffold	Putative fatty acyl	*m/z* precursor ion	Described in	Present in
Dihydro-copepodamides (*m/z* product ion: 432)	14:0	660.5	Grebner 2019	B
	15:1	672.5	This study	F
	15:0	674.5	This study	B
	16:4	680.5	This study	M
	16:3	682.5	This study	M
	16:2	684.5	This study	B
	16:1	686.5	This study	B
	16:0	688.6	Grebner 2019	B
	17:1	700.5	This study	F
	17:0	702.7	This study	F
	18:5	706.5	Grebner 2019	M
	18:4	708.6	Grebner 2019	B
	18:3	710.6	Grebner 2019	B
	18:2	712.6	Grebner 2019	B
	18:1	714.6	Grebner 2019	B
	20:5	734.6	Grebner 2019	B
	20:4	736.6	Grebner 2019	F
	20:3	738.7	This study	F
	20:1	742.6	This study	M
	22:6	760.6	Grebner 2019	B
	22:5	762.6	Grebner 2019	F
Copepodamides (*m/z* product ion: 430)	14:0	658.5	Grebner 2019	M
	16:4*	678.5	Grebner 2019	M
	16:1	684.4	Grebner 2019	M
	16:0	686.6	Grebner 2019	M
	18:5	704.5	Grebner 2019	M
	18:4	706.6	Grebner 2019	M
	18:3	708.6	Grebner 2019	M
	18:2	710.6	Grebner 2019	M
	18:1	712.6	Grebner 2019	M
	18:0	714.6	Grebner 2019	M
	20:5	732.6	Grebner 2019	M
	20:4	734.6	Grebner 2019	M
	22:6	758.7	Grebner 2019	M
	22:5	760.6	Grebner 2019	M

Putative fatty acid identity was inferred from the neutral loss associated with loss of the acyl group. Presence in marine (M), freshwater (F) or both (B) is indicated. Asterisk (*) indicate compounds found in only one sample. Grebner 2019 corresponds to reference 8 in this study.

The amount of copepodamides in the individually extracted copepods was comparable in similarly sized marine and freshwater copepods (ANCOVA: df = 1, 112, p = 0.14, η_p_^2^ = 0.019; Fig. 3e). Larger copepods contained more copepodamides than smaller ones (Linear regression: p = 0.003), but less in proportion to their mass. A millimetre sized copepod (100 µg dry weight) contained approximately 10 ng copepodamides, or 0.1 ppt of dry mass. Copepodamide content, however, varied drastically between sampling sites and individuals from F3 and F6 contained only 10–20% of the amounts found in individuals from F1-2 and F4-5 (Fig. 3e).

All bulk zooplankton samples were dominated by copepods (mean = 94% of total abundance, range = 82–100%, Supplementary Fig. S1), but freshwater samples also contained small amounts of cladocerans (0–10%) and insect larvae (0–0.5%). Similarly, marine samples also contained other zooplankton, primarily cladocerans (2–5%) and polychaete larvae (0–15%). The individually extracted freshwater copepods were dominated by cyclopoids (Supplementary Fig. S2); *Cyclops strenuus* was found in almost all samples and contributed 47% of the individually extracted freshwater copepods. Other unidentified cyclopoids were the second largest group (23%) and *Macrocyclops albidus* (2%) was only found in F4. Freshwater calanoids were only represented by *Eudiaptomus graciloides* (7%) from the ice-covered lake F6. Marine samples, in contrast, were dominated by calanoids (Supplementary Fig. S3); *Temora longicornis* was the most abundant of these (34%), followed by *Centropages hamatus* (16%) and *Calanus* sp. (16%).

**Figure 4:**
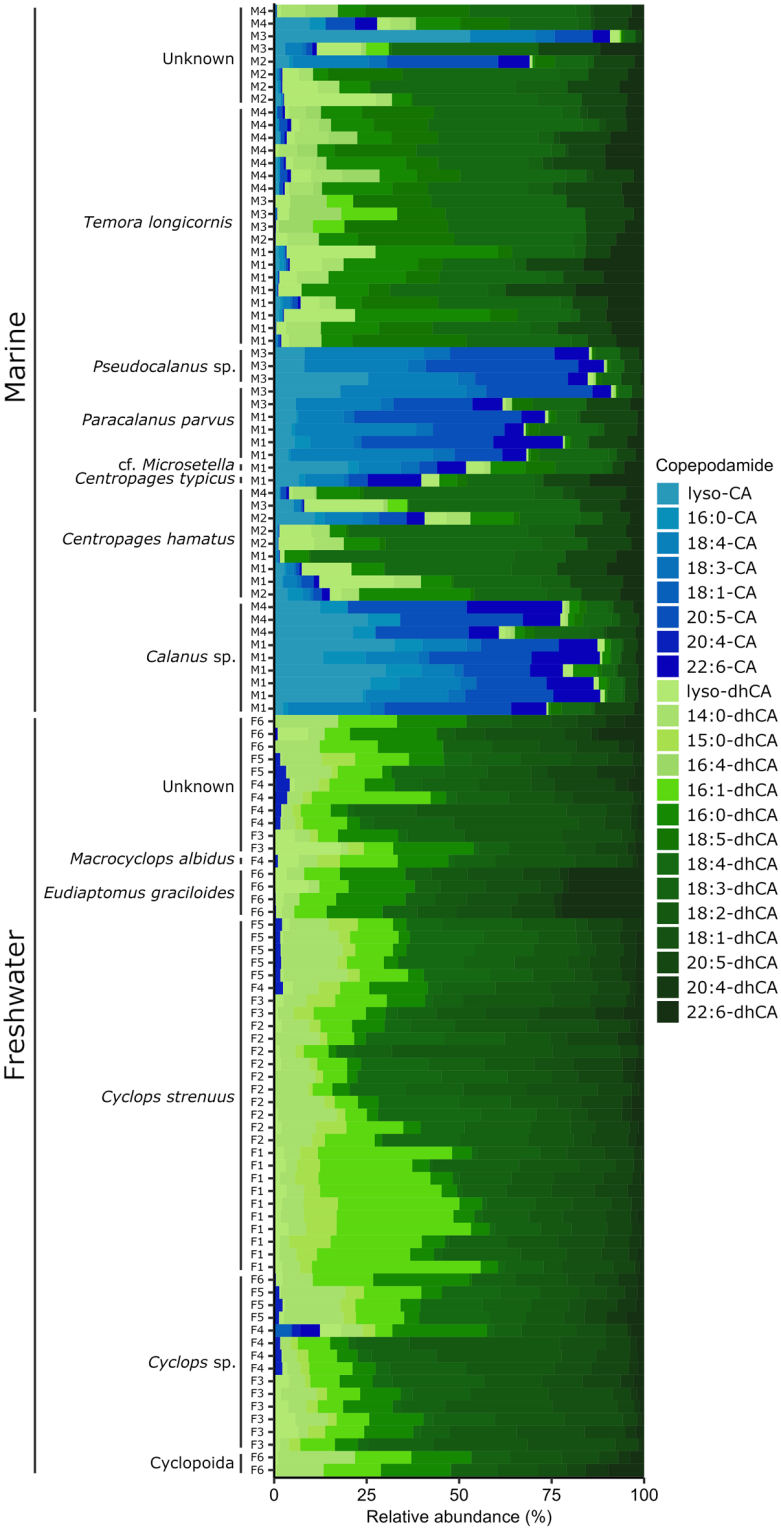
Composition of copepodamides in individual copepods from targeted LC–MS analysis of the most abundant copepodamides. Organised by habitat (marine & freshwater), taxon and sampling site. Blue and green bars denote CAs and dhCAs respectively.

The individually extracted copepods further revealed that individuals of the same taxonomic unit had similar copepodamide signatures across sampling stations and sampling times (Fig. 4). Taxonomy explained 48% of the variance left unexplained by the other model terms (partial eta squared, η_p_^2^), while habitat (freshwater or marine) explained 18%. Freshwater samples almost exclusively contained dhCAs (Figs. 3a, 4), but trace amounts of CAs were found in individual copepods from the lakes on the island Brännö (F5) and Stora Delsjön (F4). Marine samples, in contrast, contained a mixture of both copepodamide groups (Figs. 3b, 4). Table 1 contains a complete list of copepodamides with the putative identity of the acyl group inferred from the neutral loss in MS–MS experiments. Copepodamides from freshwater copepods appear to be more variable in saturation level, see for example the sequence of 18:5-, 18:4-, 18:3-, and 18:2-dhCA (Fig. 3a, Table 1). Moreover, limnic copepodamides included more odd number fatty acids such as C_15_ and C_17_. Copepodamides in marine samples, on the other hand, were dominated by polyunsaturated ω-3 fatty acids. Ten of the putative copepodamide structures found have not previously been described (Table 1). Four of these were only found in freshwater copepods.

## Discussion

We found copepodamides in all freshwater bulk zooplankton samples and in all individually extracted copepods, indicating that these compounds are also generally present in freshwater copepods. While we cannot exclude that the non-copepod zooplankton may have contributed to the copepodamide profiles in the bulk samples, the individually extracted copepod samples clearly prove the presence of copepodamides in freshwater copepods. In total we found 10 new copepodamide structures, four of which were only found in freshwater samples (Table 1). With the addition of these, a total of 41 copepodamides have been identified to date, however, the complete flat structure is known for only 10 of them. The putative structures listed here require further analyses to establish their full structure and chirality. The amounts of copepodamides were comparable to that of similarly sized marine copepods, but varied an order of magnitude between samples from different lakes. Copepods from F6 and F3 e.g., had 5–10 times lower levels of copepodamides than copepods from F1-2 and F4-5 (Fig. 3e). F6 was the only frozen and snow-covered lake we sampled from. Light penetration and, consequently, primary production are limited in these conditions^28,29^. Although the primary function of copepodamides is not known, related compounds (taurolipids) have been suggested to act as emulsifiers in lipid-uptake in the ciliate *Tetrahymena*^30^. Taurine conjugated compounds in the bile of mammals also facilitate uptake of lipids^31^. Moreover, copepodamide composition changes with the recent diet of the copepods in laboratory experiments^8^, it is therefore likely that the abundance and composition of prey species affects the copepodamide content as well. If this is the case, food-limitation may have contributed to the low amounts of copepodamides in F6. The reason for the low levels in lake F3 is less obvious. It is possible that the recent feeding history and nutritional state of the copepods contributed to this variation as well. The half-life of copepodamides in seawater is short, approximately 3 h at 15 °C^11^ but longer, around 35 h when bound in mussel tissue^18^. Differences in the handling time from sampling to extraction may consequently contribute to the observed differences. However, samples from F6 and F3 were processed after 2.4 and 3.3 h, which is longer than F1 and F2 (0.9 and 1.9 h) but not F5 (3.1 h) which was the lake with the highest copepodamide content per copepod. Moreover, the slight decrease in size-adjusted copepodamide content as a function of time was non-significant (Linear regression: p = 0.062, Supplementary Fig. S4 and Table S4). This suggests that the variation in copepodamide content was likely due to differences in phylogeny, size, or differences in food availability, rather than to processing time.

Copepodamide composition in marine and freshwater copepods clearly overlaps, as approximately one third of the copepodamides were found in both marine and freshwater copepods. Yet, the remaining two thirds were restricted to either marine or limnic copepods. Copepodamides from freshwater copepods were dominated by dhCAs, with low levels of CAs detected only in F4 and F5. F5 is situated on an island and is possibly more affected by the ocean than the other limnic systems, e.g., in terms of salt deposition. Lake F4, in contrast, is located 12 km from the sea and likely fully limnic. dhCAs are an order of magnitude more potent toxin inducers than CAs in the dinoflagellate *Alexandrium minutum*^9^, the higher proportion of dhCAs in limnic copepods may therefore be of ecological significance in freshwater environments. The identity of the fatty acyl moiety in position C5 changes with diet within days in the marine copepod *Temora longicornis,* but the ratio of dhCAs to CAs appears more stable^8^. From an ecological point of view, the partitioning between dhCAs and CAs is likely more important than differences in the fatty acid moiety which, as long as there is a fatty acid attached, seems to be of less importance for the structure–activity relationship^9,11^. The saturation level of the fatty acid moiety appears more variable in limnic samples, and these also included more odd number fatty acids such as C_15_ and C_17_ commonly found in prokaryotes^32^. This could be due to higher input of allochthonous material in limnic compared to marine environments, fuelling heterotrophic bacteria. The inherent difference in fatty acid composition in marine and limnic systems is probably the main reason for the discrepancy between marine and freshwater copepodamides^33^. C_18_ fatty acids with different degrees of saturation are common in limnic seston^34^, and the relative abundance changes with decreasing salinity in e.g., the green algae *Chlorella vulgaris* and *Acutodesmus obliquus*^35^. Future studies should include environmental data to clarify their possible effect on copepodamide composition.

The copepodamide composition is closely related to the taxonomic affinity and appears relatively stable across locations and time points (Fig. 4). This is surprising considering the rapid dynamics of the phytoplankton community composition in time and space, suggesting that copepodamide profile is stabilised either by selective feeding or species-specific differences in lipid metabolism which preserve differences between taxa. Freshwater copepods were dominated by cyclopoids and marine copepods by calanoids, this phylogenetic bias may have contributed to the differences in copepodamide composition. Copepods are believed to have evolved in the ocean and subsequentially colonised continental freshwater habitats several times^36,37^. It is possible that the identity of the colonising copepods has influenced copepodamide profiles in freshwater copepods and a more detailed phylogenetic analysis could reveal if this is the case. However, the freshwater calanoid evaluated here (*Eudiaptomus graciloides*) clearly clustered with the freshwater cyclopoids rather than the marine calanoids (Fig. 3d), suggesting that the difference in composition is not only the result of phylogenetic bias.

In conclusion we find that copepodamides are common in freshwater copepods. There is a substantial overlap with marine copepods, yet limnic copepodamides are distinct in terms of the near complete dominance of dhCAs and a different composition of fatty acyl groups. The generality of copepodamides as alarm signals in the ocean suggest that their possible role as kairomones should be studied in freshwater environments as well.

## Supplementary Information

Supplementary Information 1.

Supplementary Information 2.

Supplementary Information 3.

Supplementary Information 4.

## Data Availability

All data and analysis code generated or used in this article is stored in a public Zenodo respiratory (DOI: 10.5281/zenodo.8047944).

## Code availability

The R code used for this article, and the output it generated, is available as a HTML-file (Supplementary Code). This, and the source R-markdown file, is also available in the public repository linked to this article^27^, see section “Data Availability” for details.
