# Author Correction: Mass spectroscopy reveals compositional differences in copepodamides from limnic and marine copepods

**DOI:** 10.1038/s41598-024-83822-5

**Published:** 2025-01-09

**Authors:** Sina Arnoldt, Milad Pourdanandeh, Ingvar Spikkeland, Mats X. Andersson, Erik Selander

**Affiliations:** 1 Department of Marine Sciences, University of Gothenburg, Medicinaregatan 7B, 41390 Gothenburg, Sweden; 2 Department of Haldenvassdragets Kanalmuseum, Østfold Museum Foundation, Gamlebygata 8, 1721 Sarpsborg, Østfold, Norway; 3 Department of Biological and Environmental Sciences, University of Gothenburg, Medicinaregatan 7B, 41390 Gothenburg, Sweden; 4 Department of Biology‑Aquatic Ecology, Lund University, Sölvegatan 37, 22362 Lund, Sweden

Correction to: *Scientific Reports*, 10.1038/s41598-024-53247-1, published on 7 February 2024

The original version of this Article contained an error in Fig. 1, where the overlaid text “R^1^ = methyl (dhCA) or methylene group (CA)” was incorrectly given as “R^1^ = -methyl (CA) or -methylene group (dhCA)”. The original Fig. 1 as well as accompanying legend appear below.

**Fig. 1:**
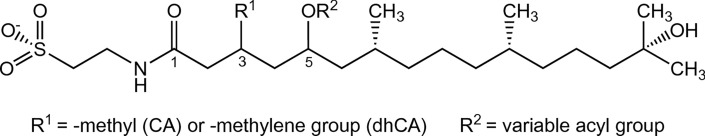
General structure of copepodamides. Two main subgroups exist, determined by the presence of methylene (copepodamide/CA) or methyl (dihydro-copepodamide/dhCA) at R^1^. The blend is species specific, but the fatty acid side chain (at position R^2^) changes with diet. Copepodamides are named by the acyl group^8^ followed by the scaffold name e.g. 22:6 dihydro-copepodamide for a dhCA scaffold with a docosahexaenoic acyl group in position R^2^.

The original Article has been corrected.

