# Supplementary material for: Mass spectroscopy reveals compositional differences in copepodamides from limnic and marine copepods
Source: Sci Rep. 2024 Feb 7;14:3147. doi: 10.1038/s41598-024-53247-1 (PMC10850141; doi:10.1038/s41598-024-53247-1)
Supplement: Supplementary file 4 — Supplementary Information 4. [file 41598_2024_53247_MOESM4_ESM.pdf]

Supplementary Information

Article title: Mass spectroscopy reveals compositional differences in chemical cues between limnic and marine copepods

Authors: Sina Arnoldt[1+], Milad Pourdanandeh[1+], Ingvar Spikkeland[2], Mats X. Andersson[3], Erik Selander[4*]

1: Department of Marine Sciences, University of Gothenburg, Medicinaregatan 7B, SE41390, Gothenburg, Sweden
2: Department of Haldenvassdragets Kanalmuseum, Østfold Museum Foundation, Gamlebygata 8, 1721, Sarpsborg, Østfold, Norway
3: Department of Biological and Environmental Sciences, University of Gothenburg, Medicinaregatan 7B, SE41390, Gothenburg, Sweden
4: Department of Biology - Aquatic Ecology, Lund University, Sölvegatan 37, SE22362, Lund, Sweden
+ Shared first authors
* Corresponding author, email: erik.selander@biol.lu.se

Public repository containing all source datasets, analysis datasets, code, and output: (DOI) 10.5281/zenodo.8047944

Table S1. Description of sampling sites.

Site	Habitat	Name of Location	Coordinates (WGS84)	Type	Sampling	Description
F1	Limnic	Botanical Garden, Gothenburg	57.677273 11.953868	Artificial pond	65µm net, 15 cm diameter, 1-2 metre depth horizontal/oblique tow	A small artificial pond in the Gothenburg Botanical Gardens, approximately 30-40 m^2, depth of 1-2 m
F2	Limnic	Trindemossen, Änggårdsbergen, Gothenburg	57.665891 11.965299	Natural lake	65µm net, 15 cm diameter, 1-2 metre depth horizontal/oblique tow	A small lake in Änggårdsbergen, Gothenburg, approximately 7 200 m^2, unknown maximum and average depth, high productivity, clear water
F3	Limnic	Finnsmossen, Änggårdsbergen, Gothenburg	57.675041 11.952768	Natural lake	65µm net, 15 cm diameter, 1-2 metre depth horizontal/oblique tow	A small lake in Änggårdsbergen, Gothenburg, approximately 12 500 m^2, unknown maximum and average depth, high productivity, low visibility,
F4	Limnic	Stora Delsjön, Gothenburg	57.680699 12.045753	Natural lake	65µm net, 15 cm diameter, 1-2 metre depth horizontal/oblique tow	One of the larger lakes in Gothenburg, popular for recreational activities. Area: 1.2 km^2. Depth: 5.2 m (average), 23 m (max). Good ecological status, high nutrient conditions, poor oxygenation in some parts, fair chemical status (except for levels of Hg and PBDEs). Source: WISS database, Swedish Water District Authorities and County Administrative Boards (https://viss.lansstyrelsen.se/)
F5	Limnic	Valmossen, Brännö	57.643242 11.773005	Natural lake	65µm net, 15 cm diameter, 1-2 metre depth horizontal/oblique tow	Small natural lake on the island Brännö outside Gothenburg. Approx. 7 400 m^2, unknown depth, high productivity
F6	Limnic	Torneträsk, Abisko	68.361747 18.802152	Natural lake	25µm net, 15 cm diameter, 1-2 metre depth horizontal/oblique tow	One of Sweden's largest lakes, located in Kiruna municipality in the north of Sweden. Area: 330 km^2, Depth: 51.8 m (average), 168 m (max), good ecological status, high nutrient levels, fair chemical status (except for levels of Hg and PBDEs). Sources: Swedish Meteorological and Hydrological Institute (https://www.smhi.se/data/hydrologi/svenskt-vattenarkiv) & WISS database, Swedish Water District Authorities and County Administrative Boards (https://viss.lansstyrelsen.se/)
M1	Marine	Gullmarn Fjord, Fiskebäckskil	58.318446 11.536843	Fjord	90µm net, 57 cm diameter, vertical tow from 25 m depth to surface, onboard R/V Oscar von Sydow	Gullmarn Fjord, between Torseröd and Alsbäck Environmental data from nearest monitoring station (SLÄGGÖ): SST = 3.7 °C, surface salinity = 21.6, Chlorophyll a = 4.6 µg/L
M2	Marine	Kristineberg, Fiskebäckskil	58.249515 11.446819	Near harbour	65µm net, 15 cm diameter, 1-2 metre depth horizontal/oblique tow	Close to the harbour of Kristineberg Center for Marine Research and Innovation. Environmental data from nearest monitoring station (SLÄGGÖ): SST = 3.7 °C, surface salinity = 21.6, Chlorophyll a = 4.6 µg/L
M3	Marine	Koster Fjord, Strömstad	58.880802 11.099231	Fjord	200 µm net, x cm diameter, vertical tow from ~50m to surface, onboard a small outboard motorised boat.	Deep water (~200 m) site between Saltö and the Koster Islands Environmental data from nearest monitoring station (SLÄGGÖ): SST = 4.4 °C, surface salinity = 30.7, Chlorophyll a = 4.4 µg/L
M4	Marine	Tjärnö, Strömstad	58.875884 11.145563	Shallow cove, near harbour	65µm net, 15 cm diameter, 1-2 metre depth horizontal/oblique tow	Shallow (5-10 m), close to the harbour of Tjärnö Marine Laboratory. Environmental data from nearest monitoring station (SLÄGGÖ): SST = 4.4 °C, surface salinity = 30.7, Chlorophyll a = 4.4 µg/L

Table S2. Number of copepods sorted from each sites bulk zooplankton sample (N sorted) and the total number of copepods in the bulk sample (Total copepods) based on either a full count or estimated by sub-sampling (*).

Site	N sorted	Total copepods
F1	10	71
F2	10	420
F3	10	285
F4	10	202
F5	10	181
F6	10	1660 *
M1	23	7840 *
M2	9	8100 *
M3	11	750 *
M4	13	2660 *

Table S3. Average estimates of terms used to estimate dry mass (g) from prosome length for different copepod taxa. Collated from published literature (below conversion equation) and averaged for the full range of prosome lengths measured in the primary sources for each taxon. Detailed calculations and regression plots are available in the public repository linked to this publication (File name: Masterfile_targeted_data_final.xlsx, Sheets: E-H).

**Taxa**	**a**	**b**
Cyclopoida	6.614E-06	2.465
Calanoida	2.801E-05	3.079
Temora	3.130E-05	3.060
Micosetella	2.239E-10	1.150
Grand mean (used for unkown taxa)	1.731E-04	2.798

Dry mass (g) = **a** x prosome length(mm)^**b**

Taxa	Source
Acartia clausi	Hay 1991
Calanus finmarchicus	Hay 1991
Calanus helgolandicus	Hay 1991
Metridia lucens	Hay 1991
Centropages typicus	Hay 1991
Centropages hamatus	Breteler et al. 1982 - Growth and devolpment of four calanoid copepod species under experimental and natural conditions
Paracalanus parvus	Hay 1991
Pseudocalanus elongatus	Hay 1991
Acartia clausi	Breteler et al. 1982
Temora longicornis	Hay 1991
Aanthocyclops vernalis	Rosen 1981 - Length-Dry Weight Relationships of Some Freshwater Zooplankton
Mesocyclops edax	Rosen 1981
Tropocyclops prasinus	Castillo-Noll 2007 - Length-weight relationships for zooplanktonic species of a tropical Brazilian lake: Lake Monte Alegre.
Cyclopoida (inc. females with eggs)	Dumont et al 1975 - The Dry Weight Estimate of Biomass in a Selection of Cladocera, Copepoda and Rotifera from the Plankton Periphyton and Benthos of Continental Waters
Cyclopoida (excl. females with eggs)	Dumont et al 1975
Pooled cyclopoida	Burgis (in press) in Dumont et al 1975 - The Dry Weight Estimate of Biomass in a Selection of Cladocera, Copepoda and Rotifera from the Plankton Periphyton and Benthos of Continental Waters
Cyclopoida 3 species C1-C6	Nakamura et al 2017 - Length-weight Relationships and Chemical Composition of the Dominant Mesozooplankton Taxa/species in the Subarctic Pacific, with Special Reference to the Effect of Lipid Accumulation in Copepoda
Tropocyclops prasinus	Castillo-Noll 2007
Microsetella norvegica	Satapoomin 1999 - Carbon content of some common tropical Andaman Sea copepods

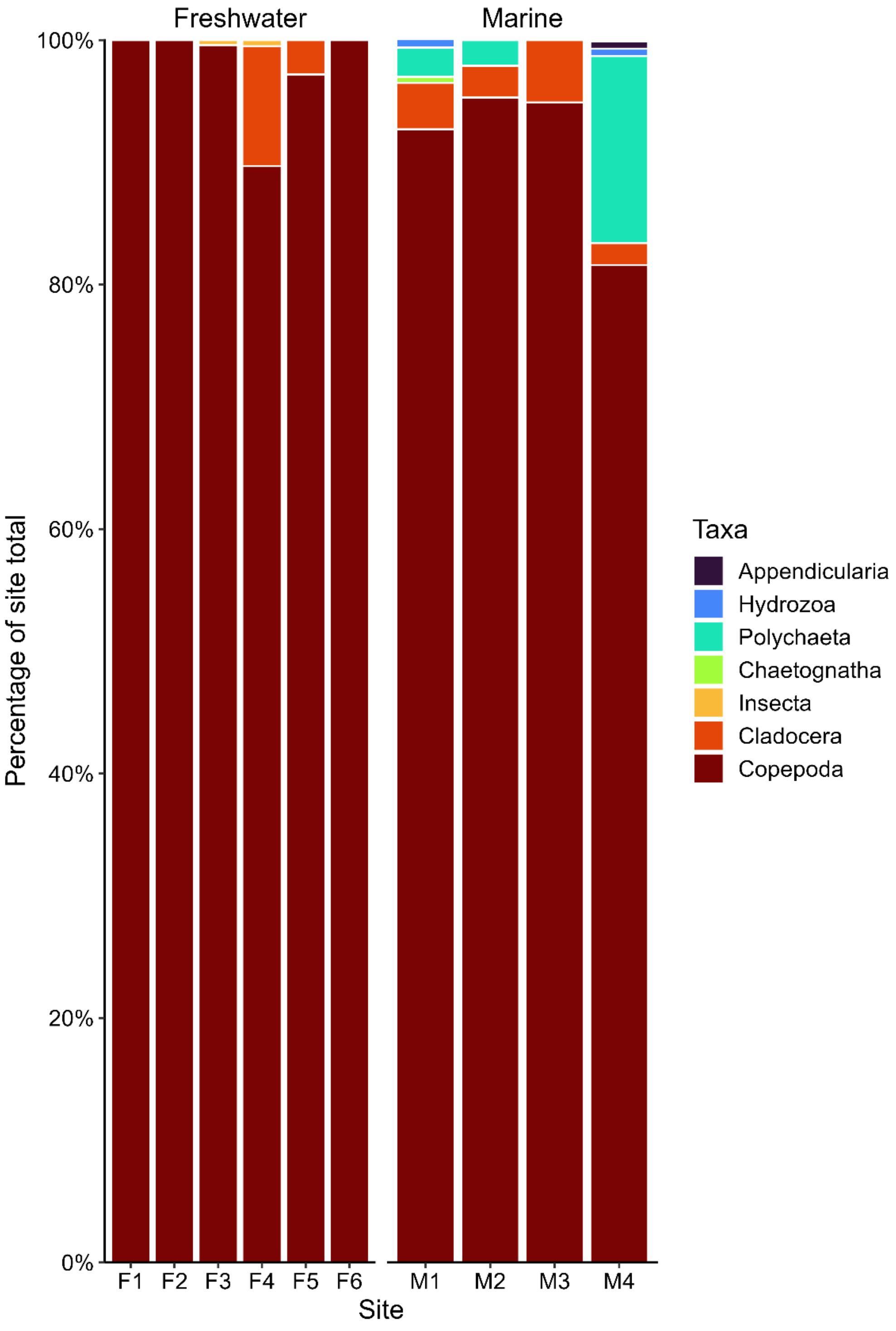

Figure S1. Composition of zooplankton in bulk samples as percentage of total abundance (F1 = 71, F2 = 420, F3 = 286, F4 = 224, F5 = 186, F6 = 1660*, M1 = 8460*, M2 = 8500*, M3 = 790*, M4 = 3260*). Asteriskt denote samples that were sub-sampled before counting, the rest (F1-F5) were counted in full.

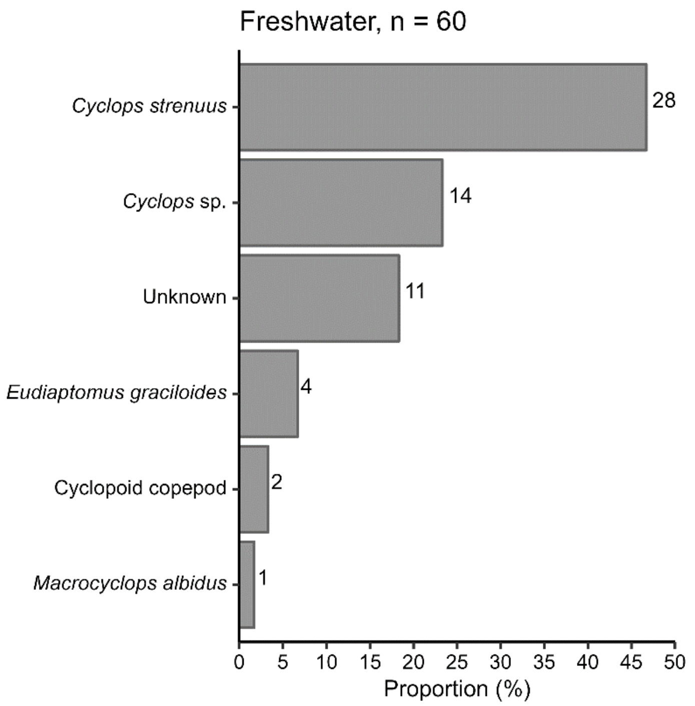

Figure S2. Taxonomic composition (%) of n = 60 individual copepods, sampled from six freshwater sites in Sweden. Values next to bars are counts of each group.

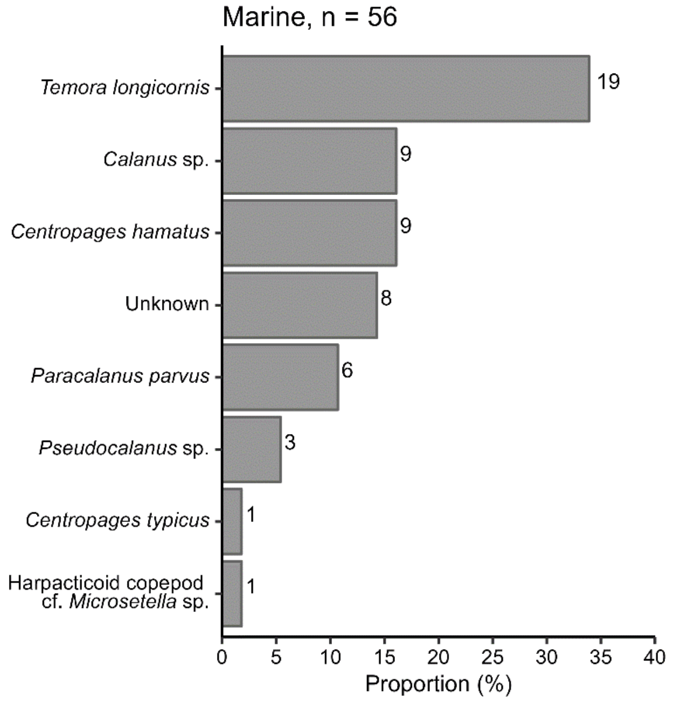

Figure S3. Taxonomic composition (%) of n = 56 individual copepods, sampled from four marine sites in Sweden. Values next to bars are counts of each group.

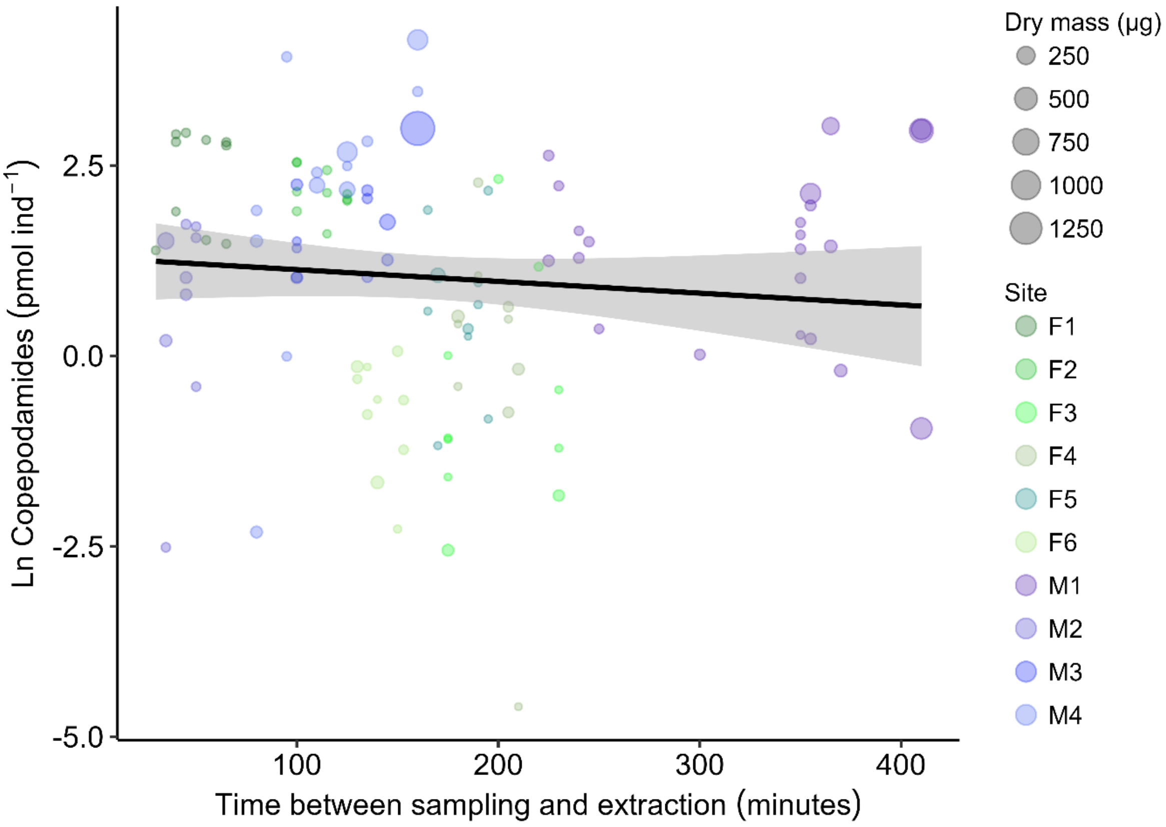

Figure S4. Relationship between copepodamide content (on a natural log scale) of individual copepods and the time between sampling and extraction. Points are coloured by sampling site (greens = freshwater , blues/purples = marine) and size scaled by the dry mass of the copepod. The black line is the least square regression line (y = -0.002x + 1.33, R^2 = 0.014) and the shaded band is the 95% confidence interval of the regression line.

Table S4. Linear regression models of copepodamide content in individual copepods (on a natural log scale) as a function of the time between sampling and extraction, adjusted for the dry mass of the copepods (left) and non-adjusted (right).

	Corrected for copepod mass			Raw time effect		
**Variable**	**Beta**	**95% CI**[1]	**p-value**	**Beta**	**95% CI**[1]	**p-value**
**Intercept**	1.3378	0.7712, 1.9044	**<0.001**	1.3294	0.7459, 1.9130	**<0.001**
**Time (min)**	-0.0029	-0.0059, 0.0001	0.062	-0.0020	-0.0050, 0.0011	0.2
**Dry mass (μg)**	0.0026	0.0008, 0.0044	**0.006**			
R^2	0.079			0.014		
df	2			1		
Residual df	113			114		

[1] CI = Confidence Interval
